# Evaluation of the Phenolic Profile of *Castanea sativa* Mill. By-Products and Their Antioxidant and Antimicrobial Activity against Multiresistant Bacteria

**DOI:** 10.3390/antiox9010087

**Published:** 2020-01-20

**Authors:** Vanessa Silva, Virgílio Falco, Maria Inês Dias, Lillian Barros, Adriana Silva, Rosa Capita, Carlos Alonso-Calleja, Joana S. Amaral, Gilberto Igrejas, Isabel C. F. R. Ferreira, Patrícia Poeta

**Affiliations:** 1 Microbiology and Antibiotic Resistance Team (MicroART), Department of Veterinary Sciences, University of Trás-os-Montes and Alto Douro (UTAD), 5000-801 Vila Real, Portugal; vanessasilva@utad.pt (V.S.); adrianaa.silva95@gmail.com (A.S.); 2 Department of Genetics and Biotechnology, University of Trás-os-Montes and Alto Douro (UTAD), 5000-801 Vila Real, Portugal; gigrejas@utad.pt; 3 Functional Genomics and Proteomics Unit, University of Trás-os-Montes and Alto Douro (UTAD), 5000-801 Vila Real, Portugal; 4 Associated Laboratory for Green Chemistry (LAQV-REQUIMTE), University NOVA of Lisboa, Lisboa, 5000-801 Caparica, Portugal; 5 Chemistry Research Centre (CQ-VR), University of Trás-os-Montes and Alto Douro (UTAD), 5000-801 Vila Real, Portugal; vfalco@utad.pt; 6 Centro de Investigação de Montanha (CIMO), Instituto Politécnico de Bragança, 5300-253 Bragança, Portugal; maria.ines@ipb.pt (M.I.D.); lillian@ipb.pt (L.B.); jamaral@ipb.pt (J.S.A.); 7 Department of Food Hygiene and Technology, Veterinary Faculty, University of León, E-24071 León, Spain; rosa.capita@unileon.es (R.C.); carlos.alonso.calleja@unileon.es (C.A.-C.); 8 Institute of Food Science and Technology, University of León, E-24071 León, Spain; 9 REQUIMTE-LAQV, Department of Chemical Sciences, Pharmacy Faculty, University of Porto, 4050-313 Porto, Portugal

**Keywords:** phenolic compounds, industrial by-products, chestnut, antioxidant capacity, antimicrobial activity, multidrug-resistant bacteria

## Abstract

The chestnut industry generates a large amount of by-products. These agro-industrial wastes have been described as potential sources of phenolic compounds with high bioactive potential. Therefore, we aimed to extract the phenolic compounds from chestnut by-products and assess their antioxidant potential and evaluate their antimicrobial activity against multidrug resistant bacteria. The individual phenolic compounds in the ethanolic extracts of chestnut shell, inner shell, bur, and leaves were characterized by HPLC-DAD/electrospray ionization (ESI)-MS. The antioxidant properties were determined by DPPH and ABTS assays. The minimum inhibitory concentration (MIC) and the antimicrobial susceptibility was performed using the Kirby–Bauer disc diffusion method against 10 bacterial strains. The major phenolic compounds identified in the extracts were trigalloyl-HHDP-glucose, gallic acid, quercetin, and myricetin glycoside derivatives. All chestnut by-products presented promising antioxidant activity in both assays, with leaf samples the ones presenting the highest antioxidant capacity. The inner shell’s extract was effective against all Gram-positive and two Gram-negative bacteria; nevertheless, all extracts showed antibacterial activity. *Staphylococcus epidermidis* showed susceptibility to all extracts while none of the extracts was able to suppress the growth of *Escherichia coli* and *Salmonella enteritidis*. Chestnut by-products are a source of phenolic compounds with prominent antioxidant and antimicrobial activities. Nevertheless, further studies should be conducted to assess the correlation between phenolic compounds and the bioactivities obtained.

## 1. Introduction

The European chestnut (*Castanea sativa,* Mill.) tree has been intensively cultivated for centuries in several countries of the Mediterranean region [1]. According to the latest data, in 2017 there was an increase in chestnut production in Europe, corresponding to 151,904 tons in a total of 17 countries, among which the main producers were Italy (34.5%), Greece (23.7%), and Portugal (19.7%) [2]. In Portugal, the chestnut sector is currently evidencing significant growth momentum with investments being made in the total growth area as well as in productivity, with the most frequently cultivated varieties being Judia and Longal. Despite a part of chestnut fruit being intended for direct consumption, a large quantity of the production is used in the food industry to obtain different products, such as gluten-free flour, marron-glacé, chestnut purée, ready-to-use frozen fruits, among others [3]. In such processes, several by-products are generated, constituted mainly by the chestnut inner and outer shells, which holds about 10% to 15% of the whole chestnut weight [4]. Those residues are usually incinerated to generate energy [5]. In addition to the inner and outer shell residues, other by-products are generated from the chestnut industry during harvesting activity, namely the burs and leaves. In this last case, these wastes are left in the field after the harvesting season, which can promote the growth of insect larvae that will further lead to crop damage [3]. For this reason, some farmers choose to gather and burn the burs and leaves, which has a negative impact on the environment. Despite the fact that several food processing by-products and/or agriculture wastes are being discarded, numerous studies have been demonstrating that they can be an attractive and cheap source of bioactive compounds with high interest for the food, cosmetic, and pharmaceutical industries [6,7,8]. In this way, the use of agro-industry residues to obtain added-value molecules or products is increasingly being suggested as part of the concept of a circular economy [9,10]. Among the molecules/products that can be obtained, phenolic compounds and/or plant extracts rich in these types of compounds have been attracting increasing attention both from researchers and industry. Phenolic compounds are formed as intermediate or end products of the secondary plant metabolism, and are considered to play a role in several processes of plants, including in growth and reproduction, as well as in adaptation and survival under stressful or adverse environmental conditions, such as ultraviolet radiation, high temperatures, and attack by pathogens [11]. Around 800 phenolic compounds have been identified in several different plant species [12]. The amount and category of phenolic compounds present in plants varies with the plant species, topography, and climate, and also in each part of the plant considered, such as fruits, seeds, leaves, stems, and peels [13]. Among the four main different classes of phenolic compounds, which are phenolic acids, flavonoids, stilbenes, and lignans, flavonoids are the most abundant ones in fruits, vegetables, cereals, and beverages being naturally consumed as part of our diets [14]. Phenolic compounds contribute to the organoleptic properties of plant food, such as color, bitterness, and astringency, among others, and participate in the oxidation processes during production and conservation [15]. Besides being important for the plant, in recent years, evidence has accumulated that long-term consumption of diets rich in polyphenols may have implications for human health, specifically by reducing the incidence of cardiovascular diseases and some types of cancer [16]. Phenolic compounds have also been described as having antimicrobial, antioxidant, anti-inflammatory, anti-hepatotoxic, antidiarrheal, antiviral, anti-ulcer, antiallergic, and vasodilatory activity [17]. Most of these properties are related to their antioxidant power, their ability to modulate or inhibit topoisomerase-like enzymes, and their effect as chelating agents [18,19,20]. In particular, the antibacterial activity exhibited by some phenolic compounds has been an emerging research focus in recent years [21] due to the increasing rise of multidrug resistant bacterial strains observed in the last years, thus, the proposal of alternatives to combat bacteria that are resistant to conventional antibiotics is essential.

Nuts are foods traditionally linked to the Mediterranean diet. The regular consumption of nuts leads to the reduction of the levels of total cholesterol and LDL cholesterol in the blood and the lower oxidation rate of LDL, thus being associated with a lower incidence of cardiovascular diseases [22]. These benefic effects on human health are in part attributed to the presence of phenolic compounds and their antioxidant activity [23]. Phenols are present both in the fruit and in the different coverings of the fruit. Different works have been previously performed on chestnut industry wastes demonstrating that they are an interesting source of bioactive compounds, but they generally include only few individual components (flower, leaves, shells, or burs) and are mainly focused on the evaluation of antioxidant activity and extraction methods [4,6,7,8,24,25,26,27,28]. Thus, there is still a scarcity of studies regarding the detailed evaluation of their antimicrobial activity. Considering the current interest in searching for alternative sources of antimicrobial compounds or compounds with antibiotic resistance-modulatory effects, as well as for searching low-cost sources of natural antioxidant compounds, in the present work we aimed to extract bioactive components from chestnut industry by-products, characterize their content of phenolic compounds, and evaluate their antioxidant and antibacterial activities.

## 2. Materials and Methods

### 2.1. Plant Material and Extract Preparation

Plant material used included chestnut industry by-products, namely, the inner and outer shells, burs, and leaves of the Longal variety. Samples were collected in November 2017 from a local orchard in the north of Portugal. Chestnut components were manually separated, lyophilized, mill-powdered, and stored at −20 °C. The lyophilized powdered samples (2 g) were extracted with 100 mL of ethanol, by stirring for two hours at ambient temperature, followed by sonication for 5 min. Samples were centrifuged for 15 min at 10,000× *g* and the pellet was re-extracted. The supernatants of each extraction were collected, filtered, and the solvent evaporated on a rotary evaporator at 40 °C under reduced pressure. The dried extracts were redissolved in ethanol to a final concentration of 5 mg/mL and in dimethyl sulfoxide (DMSO) to a final concentration of 100 mg/mL, for the analysis of phenolic composition and antimicrobial activity, respectively. The extracts were stored under −20 °C until further analysis.

### 2.2. Spectrophotometry Estimation of Total Phenolic and Tannin Content

The total phenols in the inner and outer shells, burs, and leaves were determined spectrophotometrically according to Connor, Luby, and Tong (2002) [29]. Briefly, 3.8 mL of HCl 1.0 M was added to 200 µL of sample extracts and allowed to stand at room temperature for 3 h. Then, the absorbance was measured at 280 nm. DMSO was used as the reference solution and each determination was performed in triplicate.

The determination of total tannin content was performed as previously described by Sarneckis, Dambergs, Jones, Mercurio, and Herderich (2006) [30]. For each sample, 2 preparations were performed as follows: 600 µL of methyl cellulose solution (0.04%) was added to 50 µL of each sample in a 2 mL centrifuge tube. The tube was inverted a few times and was incubated at room temperature for 3 min. Four hundred microliters of saturated ammonium sulphate solution were added to the mixture and the volume was made up to 2 mL with deionized water. The solution was allowed to stand for 10 min at room temperature, after which it was centrifuged for 15 min at 10,000× *g*. The absorbance was measured at 280 nm. For the second preparation (control sample), the same procedure was followed except deionized water was used instead of methyl cellulose solution.

### 2.3. Determination of Phenolic Compounds by HPLC-DAD-Electrospray Ionization (ESI)/MS

Phenolic compounds were analyzed after the redissolution of extracts to a concentration of 5 mg/mL and filtering through a 0.22 μm disposable filter disk. The chromatographic system used for the analysis consisted of a Dionex Ultimate 3000 UPLC system (Thermo Scientific, San Jose, CA, USA) equipped with an auto-sampler operating at refrigerated temperature (5 °C), a degasser, a quaternary pump, and a diode array coupled in-series to a Linear Ion Trap LTQ XL mass spectrometer (Thermo Finnigan, San Jose, CA, USA) equipped with an electrospray ionization (ESI) source. Chromatographic separation was achieved with a Waters Spherisorb S3 ODS-2C18 (3 μm, 4.6 × 150 mm, Waters, Milford, MA, USA) column thermostatized at 35 °C. The elution conditions and detector settings were identical to those described by Bessada, Barreira, Barros, Ferreira, and Oliveira (2016) [31].

The identification of the compounds was performed by comparison of data regarding retention time, UV–Vis spectra recorded preferentially at 280 and 370 nm, mass spectra (recorded in full scan mode, covering a mass range from *m/z* 100 to 1500), and fragmentation pattern of the sample compounds with those obtained from the available standards and/or reported data from literature. The identified compounds were quantified using the calibration curves constructed based on the UV–Vis signal of the following standards, ellagic acid, gallic acid, myricetin, quercetin-3-*O*-rutinoside, and quercetin-3-*O*-glucoside (Extrasynthese, Genay, France) were used. The results were expressed in mg/g extract.

### 2.4. Antioxidant Activity

Antioxidant capacity of each sample was investigated by the ABTS (2,2-azino-di-(3-ethyl-benzothialozine-sulphonic acid)) and DPPH (2,2-diphenyl-1-picrylhydrazyl) assays. The determination of the antioxidant activity by the former method was performed as described by Re et al. (1999) [32]. Initially, the ABTS^+^ radical solution was prepared from the reaction of 7 mM ABTS with 2.45 mM potassium persulfate (final concentration) incubated at room temperature and in the absence of light for 12 h. Then, the solution was diluted in ethanol to an absorbance solution of 0.70 (± 0.01) at 734 nm. Samples were diluted in ethanol to 20–80% radical inhibition and compared to blank uptake. To perform the measurements, 40 μL of the diluted sample were added to 1960 μL of the solution containing the radical, and the absorbance was determined by spectrophotometry at 734 nm after 2, 4, 6, 10, 20, 30, and 60 min of reaction. Trolox was used as a standard solution at concentrations of 100 to 200 μM in ethanol. All determinations were performed in triplicate, and results were expressed in Trolox equivalent antioxidant capacity (TEAC µmol Trolox/g).

The determination of antioxidant capacity by the DPPH method was performed according to Hatano, Kagawa, Yasuhara, and Okuda (1998) [33]. A DPPH methanolic solution (6 × 10^−5^ M) was prepared and added to different dilutions of the sample’s extracts. The mixtures were incubated in the dark for 1 h at room temperature. After the incubation period, the reading was undertaken at 517 nm. The radical scavenging activity (RSA) was calculated as a percentage of DPPH discoloration according to the following formula: RSA% = [(ADPPH – AS)/ADPPH] × 100, in which the AS is the absorbance of the sample solution and ADPPH is the absorbance of the blank control. The result of antioxidant activity was expressed as the ability to sequester/reduce the DPPH radical, i.e., the amount of extract required to reduce the initial DPPH concentration by 50% (EC_50_). All the analyses were performed in triplicate. 

### 2.5. Antibacterial Activity

#### 2.5.1. Bacterial Strains

Antimicrobial susceptibility testing was performed against four multidrug resistant Gram-positive bacteria: *Enterococcus faecalis* vanB2-C3735 [34], *Enterococcus faecium* vanA-C2302 [35], *Staphylococcus aureus* C5932 (MRSA CC398) [36], and *Staphylococcus epidermidis* C3658 (linezo-R) [37], and four multiresistant Gram-negative bacteria: *Salmonella enteritidis* C4220, *Escherichia coli* C999 (CTX-M-15) [38], *Klebsiella pneumoniae* C1370 (CTX-M-15) [38], and *Pseudomonas aeruginosa* C4660 (VIM-2) [39]; and two Gram-positive foodborne strains *Listeria monocytogenes* ATCC700302 and *Bacillus cereus* ATCC1306. The strains are part of the University of Trás-os-Montes and Alto Douro and University of La Rioja collections. All the bacterial strains were subcultured from the original culture in brain heart infusion (BHI) agar (Oxoid, Basingstoke, UK) for 24 h at 37 °C. Müller–Hinton (MH) agar (Oxoid, Basingstoke, UK) was used for the antimicrobial susceptibility assay.

#### 2.5.2. Antimicrobial Susceptibility Test

The antimicrobial susceptibility assay was performed using the Kirby–Bauer disc diffusion method. The measurement of bacterial growth inhibition was carried out as previously described [40]. Each bacterial strain was seeded in BHI agar plates and incubated overnight at 37 °C. Colonies were suspended in physiological solution to a turbidity equivalent to 0.5 McFarland standard and inoculated on MH plates. The extract solution of 100 mg/mL in DMSO was used for the initial evaluation of antimicrobial susceptibility. The initial concentration was further diluted with DMSO to 75, 50, 25, and 10 mg/mL and tested against bacteria that showed susceptibility to the extracts in the initial assay. Twenty microliters of each extract concentration were loaded on sterile blank discs (6 mm diameter) and the discs were placed on the inoculated MH plates, which were incubated for 24 h at 37 °C. The inhibition zones were measured with a ruler, recorded, and considered as indication for antibacterial activity. Discs loaded with DMSO were used as the negative control. Antibiotic discs loaded with chloramphenicol *(S. aureus, S. epidermidis, E. coli, K. pneumoniae,* and *B. cereus)*, trimethoprim/sulfamethoxazole (*E. faecalis, E. faecium,* and *L. monocytogenes*) and ciprofloxacin (*P. aeruginosa*) were used as the positive control. The assay was performed in duplicate.

### 2.6. Statistical Analysis

The results were expressed as mean values and standard deviation (SD) and analyzed using one-way analysis of variance (ANOVA) followed by Tukey’s HSD test with *p* = 0.05. Levene’s test was performed to verify if there was homogeneity of variances. For the individual phenolic compound quantification, a Student’s t-test was used to determine the significant difference, with *p* = 0.05. The analyses were carried out using IBM SPSS Statistics for Windows, Version 26.0 (IBM Corp., Armonk, New York, NY, USA).

## 3. Results and Discussion

### 3.1. Phenolic Compounds

Despite allowing only the estimation of the content of the group of compounds, the use of spectrophotometric methods presents several advantages over chromatographic ones, namely the cost, fastness, and not requiring such a high level of expertise of the technician performing the analysis, therefore being suited for a screening approach that can be carried out even by small laboratories in industries. Therefore, as a first approach, the total phenolics content (TPC) and total tannin content (TTC) of the extracts prepared with the chestnut industry by-products, namely, the inner and outer shells, bur, and leaves, were estimated by spectrophotometry, as shown in Table 1. Among all by-products, the leaves had the highest phenolic content (385.4 ± 0.5 µg of epicatechin equivalents (EE)/mg of residue), followed by the inner shell (321 ± 3 EE/mg), the bur (242 ± 1 EE/mg), and the outer shell (240 ± 6 EE/mg). The extraction efficiency for TPC, as indicated by this approach, may be synthesized as: leaves > inner shells ≫ bur > outer shells. Similar to these results, Vella et al. (2018) used different solvents to extract polyphenols from chestnut industry wastes and reported that the extracts from chestnut leaves were the ones with the highest TPC, with the shells presenting a very low phenolic content when compared to burs and leaves. In the present study, the TPC of burs and outer shells was quite similar, while the TPC of inner shells was comparable to the leaves. Regarding the estimation of tannin content, TTC was higher on the leaves (113 ± 1 EE/mg), followed by the inner shell (35 ± 5 EE/mg), and was quite low in the outer shell (9 ± 1 EE/mg) and the bur (5.5 ± 0.4 EE/mg). As previously reported, tannins are present in higher concentrations in the inner shell and are associated with the bitter taste found in this chestnut part [41]. Previously, Živković et al. (2009) evaluated the TTC of chestnut leaves, catkins, seed, bark, and spiny burs, and reported a higher TTC in the inner shell [42]. Nevertheless, and in accordance with our results, Vella et al. (2018) have found a higher TTC on chestnut leaves compared to those of the shells or burs [4].

In order to obtain more insights on the qualitative profile of the chemical composition of the prepared chestnut by-product extracts, those were further evaluated by HPLC-DAD-ESI/MS. The results of the phenolic composition of chestnut inner and outer shells, bur, and leaves are shown in Table 2. Overall, 14 different compounds were identified and quantified. The inner shell presented four compounds (three flavonol glycoside derivatives and a phenolic acid), while the outer shell revealed two compounds (one phenolic acid and one flavonol glycoside derivative), the bur presented six compounds (five flavonol glycoside derivatives and an ellagitannin), and in the leaves, nine compounds were tentatively identified (eight flavonol glycoside derivatives and a ellagitannin). Gallic acid (peak 1), myricetin-3-*O*-glucoside (peak 3), quercetin-3-*O*-rutinoside (peak 4), quercetin-3-*O*-glucoside (peak 7), ellagic acid (peak 8), kaempherol-3-*O*-rutinoside (peak 9), isorhamnetin-3-*O*-rutinoside (peak 10), and quercetin-3-*O*-rhamnoside (peak 11), were positively identified in comparison with the DAD and MS spectrums of the commercial phenolic compounds standards (Extrasynthese, Genay, France). Other flavonol glycoside derivatives identified were peaks 5 ([M-H]^−^ at *m/z* 463) and 6 ([M-H]^−^ at *m/z* 477), which are coherent to myricetin and quercetin glycoside derivatives, due to the UV-Vis spectra (λmax around 350 nm) and the MS^2^ fragment at *m/z* 317 and 301, respectively. Both compounds presented a loss of a deoxyhexoside (−146 u), thus the exact sugar moiety and position on the aglycone could not be established, thus being tentatively identified as myricetin-*O*-deoxyhexoside and quercetin-*O*-deoxyhexoside. Similarly, peak 13 ([M-H]^−^ at *m/z* 477) and 14 ([M-H]^−^ at *m/z* 507) were tentatively assigned as isorhamnetin-*O*-pentoside and syringetin-*O*-hexoside. Due to its UV-Vis spectra and MS^2^ fragment at *m/z* 285, peak 12 ([M-H]^−^ at *m/z* 739) was assigned as a kaempferol derivative. It presented two fragments at *m/z* 593 (−146 u) and 285 (−308 u), which could correspond to the loss of a *p*-coumaroyl (due to its low UV-Vis spectra) and rutinosyl units, being tentatively identified as kaempferol-O-(*p*-coumaroyl)-rutinoside. Many of the mentioned compounds have been previously identified in *C. sativa* flowers [43,44], burs [6,8,25,45], leaves [46], and heartwood [47,48]. The only ellagitannin found corresponded to peak 2 ([M-H]^−^ at *m/z* 937) and was identified as a trigalloyl-HHDP-glucose, also known as chestanin A, with a characteristic MS^2^ fragment at *m*/*z* 301 (ellagic acid) and suffered the loss of gallic acid (*m/z* 767) and the fragments *m/z* 635 and 465 were ascribed to the loss of hexahydroxydiphenoyl (HHDP) and gallic acid. The HHDP group is released during acid hydrolysis and lactonizes to ellagic acid [49].

Leaves were the chestnut part that revealed the highest individual phenolic content, with trigalloyl-HHDP-glucose (18.0 ± 0.5 mg/g) as the main compound, followed by the outer shells in which gallic acid was the main constituent, and the bur presenting trigalloyl-HHDP-glucose (1.80 ± 0.02 mg/g) as the main molecule. The inner shell was the chestnut part that presented the lowest phenolic compounds, being syringetin-*O*-hexoside as the main molecule. The main phenolic compounds reported to be present in chestnut by-products are phenolic acids, in particular ellagic and gallic acids, flavonoids (quercetin derivates), and tannins [50]. Trigalloyl HHDP derivates have been previously reported as part of chestnut by-products. Studies analyzed the phenolic composition of chestnut flowers in Judia and Longal cultivars [44] and heartwood [47] and found this compound to be the most abundant flavonoid, in particular, its derivates trigalloyl HHDP glucose and trigalloyl HHDP glucoside [43,44,51]. Furthermore, one should refer that trigalloyl HHDP glucose seems to be very common in nuts in general [49,52,53]. It is widely known that sweet chestnut (*Castanea sativa* Mill.) and its by-products are rich in ellagitannins that form hexahydroxydiphenic (HHDP) acid which, in turn, by the *intra*-molecular esterification reaction creates the ellagic acid [51]. Quercetin, isorhamnetin, and kaempherol derivates have been mainly found in chestnut leaves and bur extracts, which is in accordance with other studies and have been described as compounds with antioxidant proprieties [45]. A study made by Vázquez et al. (2012) in chestnut bur extracts demonstrates the contribution of phenolic compounds, as gallic acid esters of glucose and ellagic acid, to antioxidant activity of the extracts [54]. Despite the fact that the shells of the chestnut are well-known for being rich in tannin, this study was in agreement once inner shells presented a high content of tannins, and ellagic acid was detected in inner shells at low concentrations [54]. All myricetin derivatives were detected in the inner shell, which is in good agreement with previous studies conducted with chestnut flowers that reported the presence of several myricetin derivatives in ethanolic extracts [43]. Chestnut shells’ phenolic contents are described as the major contributors to the antioxidant properties of the extracts [43].

### 3.2. Antioxidant Activity

In this study, the antioxidant activity of the ethanolic extracts of chestnut inner and outer shells, bur, and leaves were evaluated using the two different screening assays, ABTS and DPPH, with the obtained results being shown in Table 3. The results of the ABTS assay were in accordance with those obtained in the DPPH assay. As expected, the antioxidant activity increased with the TPC, as chestnut leaves presented a higher antioxidant power for both assays, followed by the inner shells, bur, and outer shells. Furthermore, the same was observed for the TTC. Despite better results being obtained for the leaves, in general, all chestnut by-products presented promising antioxidant activity, both in the ABTS and DPPH assays. Overall, all extracts displayed considerably low IC_50_ values and were comparable to the results obtained previously by other authors [4,24]. Nevertheless, when evaluating the same chestnut variety (Longal), Barreira, Ferreira, Oliveira, and Pereira (2008) reported a higher antioxidant activity for the chestnut shells compared to the chestnut flowers and leaves, while in the present study, better activity was obtained for the leaves. These reducing properties of shells and flowers are associated with the presence of reductones, and these chestnut by-products have a high presence of these compounds, which have been shown to exert antioxidant activities by breaking the radical chain by donating a hydrogen atom [24]. This difference may be due to the collection period, since the authors collected some of the samples in July, whereas all of our samples were collected in November. Vázquez et al. (2009) analyzed the antioxidant activities of chestnut burs using several different assays and found higher results compared to our results from bur extracts [27]. Lorenzo, González-Rodríguez, Sánchez, Amado, and Franco (2013) assessed the antioxidant activity of chestnut leaves collected in Spain and obtained less expressive results (DPPH EC_50_ 1.04 g/L), which could be related to the extraction method (aqueous extract) since other studies also carried out on chestnut by-products reported a higher antioxidant activity for ethanolic than for water extracts [26]. Nevertheless, it should be taken into account that other compounds besides phenolic compounds can potentially be present in ethanolic extracts, contributing also to antioxidant properties. Moreover, the synergistic effect of different bioactive compounds present in plants may also enhance the antioxidant properties of the whole extracts [55].

### 3.3. Antimicrobial Activity

All the chestnut by-product extracts were initially tested at a concentration of 100 mg/mL against eight multidrug-resistant bacteria isolated from different sources, and two food-borne bacteria, using the disc diffusion method. The obtained results are shown in Table 4. Regarding the Gram-positive bacteria, *S. epidermidis* and *S. aureus* presented susceptibility to all extracts, while *E. faecalis* showed susceptibility to both inner shell and bur extracts and *E. faecium* was susceptible only to the inner shell extract. As for the Gram-negative bacteria, none of the extracts had any effect on the growth of *E. coli* and *S. enteritidis*, while *P. aeruginosa* was susceptible to three of the assayed extracts and *K. pneumoniae* was susceptible only to the inner shell extract. The extracts were very effective in suppressing *S. epidermidis* growth since it presented the larger inhibition zones. From all the prepared extracts, the one from the chestnut’s inner shell was the most effective in suppressing microbial growth, since it was effective against 6 out of the 10 bacteria tested, whereas all the other extracts showed variable antimicrobial activity being effective against only 3 bacteria each. Subsequently, another experiment was conducted to determine the minimal inhibitory concentration (MIC) against the susceptible bacterial strains. The MIC values of each extract are shown in Table 5, where it is possible to observe that they ranged from 10 to 75 mg/mL, being higher in Gram-negative bacteria. Overall, the inner shell extract presented the lowest MIC values against Gram-negative bacteria, whereas the leaf extract had the lowest MIC values on Gram-positive bacteria. The antibacterial activity mechanisms of phenolic compounds have been extensively studied and it has been reported that they can act on the cell membrane, inactive essential enzymes, and/or modify the function of genetic material [56]. The chestnut extracts were not effective against *E. coli* and *S. enteritidis* and the other Gram-negative bacteria displayed small inhibition zones for the highest concentration of the extracts. Despite the fact that all Gram-negative bacteria present multiple antibiotic resistances, it is known that cell walls of Gram-negative bacteria represent a major barrier for the entry of phenolic compounds into the cell cytoplasm due to the repulsion between lipopolysaccharide found in the surfaces of Gram-negative bacteria and phenols [56,57]. Nevertheless, both *K. pneumoniae* and *P. aeruginosa* were inhibited by inner and outer shell extracts with MICs of 50 and 75 mg/mL, respectively. These results may be due to the fact that chestnut shells have phenolic acids, which are known to have an antibacterial effect against Gram-negative bacteria. Lou et al. (2012) conducted a study with isolated phenolic compounds and reported that phenolic acids, in particular *p*-coumaric acid, are effective against Gram-negative bacteria by binding to DNA or changing the permeability of the cell membrane [58]. Leaf extracts at low concentrations showed strong antibacterial activity against *S. epidermidis* and *S. aureus* resulting in large inhibition zones. In accordance with our results, Živković et al. (2010) studied the antibacterial action of chestnut by-products against several bacteria, including *S. aureus* and *B. cereus*, and reported a higher inhibition zone for the leaf extracts in comparison with the other extracts [59]. Leaves presented the highest individual phenolic content with a high concentration of the ellagitannins trigalloyl-HHDP-glucose (18.0 ± 0.5 mg/g). Furthermore, bur extracts also contained this ellagitannin and were effective against *S. epidermidis, S. aureus,* and *E. faecalis* at 25, 25, and 10 mg/mL. Ellagitannins have proven antibacterial activity against several different species of bacteria [60]. Despite the fact that only two phenolic compounds were identified in outer membrane extracts, gallic acid was found in an elevated concentration. Other studies reported the susceptibility of *S. aureus* and *Salmonella* to gallic acid, with the MIC of *S. aureus* much lower when compared to *Salmonella* [61].

## 4. Conclusions

The obtained results add evidence that chestnut industry by-products can be an interesting source of phenolic compounds with antioxidant and antibacterial activities. The ethanolic extract of chestnut leaves presented the highest content in total phenolic compounds and the highest antioxidant activity, followed by the inner shell. Despite the fact that only four phenolic compounds were identified in the inner shell extract, this was the most effective against multidrug resistant bacteria. These findings suggest that chestnut by-products may provide coadjutors to antibiotics, be used as nutraceuticals and antioxidant additives, therefore creating added economic value for the chestnut industry, which produces high quantities of the studied by-products. Furthermore, the utilization of phenolic compounds extracted from chestnut by-products has remarkable importance within the circular economy principles of production and utilization of natural resources.

## Figures and Tables

**Table 1:** Total phenol and tannin contents of chestnut by-products (mean value ± SD, *n* = 3).

Sample	Total Phenol Content *	Total Tannin Content *
**Inner shell**	321 ± 3 ^b^	35 ± 5 ^b^
**Outer shell**	240 ± 6 ^c^	9 ± 1 ^c^
**Bur**	242.4 ± 0.9 ^c^	5.5 ± 0.4 ^c^
**Leaves**	385.4 ± 0.5 ^a^	113 ± 1 ^a^

* Values expressed as µg of epicatechin equivalents/mg of residue. Different letters indicate significant differences (*p* < 0.05).

**Table 2:** Retention time (Rt), wavelengths of maximum absorption in the visible region (λ_max_), mass spectrometric data, tentative identification, and quantification (mg/g extract) of the phenolic compounds present in the ethanolic extracts of chestnut inner and outer shells, bur, and leaves.

Peak	Rt (min)	λ_max_ (nm)	[M − H]^−^ (*m/z*)	Main MS^2^ Fragments (*m*/*z*)	Tentative Identification	Inner Shell	Outer Shell	Bur	Leaves	*t*-Students Test *p*-Value
**1**	4.01	270	169	125(100)	Gallic acid	nd	8.3 ± 0.2	nd	nd	-
**2**	14.39	276	937	767(5), 637(18), 467(100), 301(5)	Trigalloyl-HHDP-glucose	nd	nd	1.80 ± 0.02	18.0 ± 0.5	<0.001
**3**	15.4	352	479	317(100)	Myricetin-3-*O*-glucoside	0.522 ± 0.002	nd	nd	nd	-
**4**	17.64	355	609	301(100)	Quercetin-3-*O*-rutinoside	nd	nd	0.474 ± 0.001	1.845 ± 0.004	<0.001
**5**	17.74	351	463	317(100)	Myricetin-*O*-deoxyhexoside	0.505 ± 0.001	nd	nd	nd	-
**6**	18.02	354	477	301(100)	Quercetin-*O*-deoxyhexoside	nd	nd	nd	1.08 ± 0.02	-
**7**	18.8	354	463	301(100)	Quercetin-3-*O*-glucoside	nd	nd	0.521 ± 0.002	2.33 ± 0.08	<0.001
**8**	19.42	362	301	256(10), 185(5)	Ellagic acid	0.289 ± 0.001	nd	nd	nd	-
**9**	21.04	347	539	285(100)	Kaempherol-3-*O*-rutinoside	nd	nd	0.493 ± 0.001	0.94 ± 0.03	<0.001
**10**	22.03	350	623	315(100)	Isorhamnetin-3-*O*-rutinoside	nd	nd	0.669 ± 0.002	0.69 ± 0.02	0.002
**11**	22.25	351	447	301(100)	Quercetin-3-*O*-rhamnoside	nd	nd	0.516 ± 0.003	1.138 ± 0.003	<0.001
**12**	22.58	314	739	593(17), 285(100)	Kaempferol-*O*-(*p*-coumaroyl)-rutinoside	nd	nd	nd	0.486 ± 0.006	-
**13**	23.09	353	447	315(100)	Isorhamnetin-*O*-pentoside	nd	nd	nd	1.97 ± 0.03	-
**14**	23.67	352	507	345(100)	Syringetin-*O*-hexoside	0.568 ± 0.001	1.112 ± 0.004	nd	nd	<0.001
					**Total Phenolic compounds**	1.883 ± 0.003^d^	9.4 ± 0.2^b^	4.48 ± 0.01^c^	28.5 ± 0.6^a^	-

nd—not detected; standard calibration curves: A—gallic acid (*y* = 131538*x* + 292163, *R^2^*= 0.9969, LOD (limit of detection) = 8.05 µg/mL and LOQ (limit of quantification) = 24.41 µg/mL, peak 1); B—ellagic acid (*y* = 26719*x* – 317255, *R²* = 0.9986, LOD = 41.20 µg/mL and LOQ = 124.84 µg/mL, peaks 2 and 8); C—myricetin (*y* = 23287*x* – 581708, *R²* = 0.9988, LOD = 61.21 µg/mL and LOQ = 185.49 µg/mL, peaks 3, 5, and 14); D—quercetin-3-*O*-rutinoside (*y* = 13343*x* + 76751, *R²* = 0.9998, LOD = 14.71 µg/mL and LOQ = 44.59 µg/mL, peaks 4, 6, 9, 10, 11, 12, and 13); E—quercetin-3-*O*-glucoside (*y* = 34843*x* – 160173, *R²* = 0.9998, LOD = 17.01 µg/mL and LOD = 51.54 µg/mL, peak 7). For total phenolic compounds, an ANOVA analysis was performed, with different letters indicating significant differences (*p* < 0.05).

**Table 3:** Antioxidant activity of the inner and outer shells, bur, and leaves of chestnut (mean value ± SD, *n* = 3).

Chestnut Component	ABTS ^a^	DPPH ^b^
**Inner shell**	3533 ± 1 ^b^	0.06 ± 0.01 ^c^
**Outer shell**	203 ± 2 ^d^	0.12 ± 0.02 ^a^
**Bur**	801 ± 5 ^c^	0.09 ± 0.01 ^b^
**Leaves**	5861 ± 5 ^a^	0.03 ± 0.01 ^d^

^a^ expressed in μmol Trolox/g of residue; ^b^ expressed in effective concentration at which 50% of DPPH radicals are scavenged (EC50, extract concentration (mg/mL); Trolox was used as a positive control, with Trolox EC50 = 0.04 ± 0.01 mg/mL). Different letters indicate significant differences (*p* < 0.05).

**Table 4:** Antimicrobial susceptibility (inhibition zones, mm) of multidrug resistant Gram-positive and Gram-negative bacteria to the ethanolic extracts from chestnut inner and outer shells, bur, and leaves.

Bacterial Strain	Inner Shell	Outer Shell	Bur	Leaves	Positive Control
**Gram-positive**					
*S. epidermidis*	18	17	12	20	30
*S. aureus*	12	10	11	15	26
*E. faecalis*	11	-	11	-	25
*E. faecium*	11	-	-	-	26
*L. monocytogenes*	-	-	-	-	34
*B. cereus*	-	-	-	-	32
**Gram-negative**					
*K. pneumoniae*	10	-	-	-	0
*E. coli*	-	-	-	-	28
*P. aeruginosa*	9	9	-	10	18
*S. enteritidis*	-	-	-	-	25

- not determined.

**Table 5:** Minimum inhibitory concentration (MIC, mg/mL) of the ethanolic extracts from chestnut inner and outer shells, bur, and leaves against multidrug resistant Gram-positive and Gram-negative bacteria.

Bacterial Strain	Inner Shell	Outer Shell	Bur	Leaves	Positive Control
**Gram-positive**					
*S. epidermidis*	25	10	25	10	<32
*S. aureus*	50	50	25	25	
*E. faecalis*	25	-	10	-	
*E. faecium*	25	-	-	-	
**Gram-negative**					
*K. pneumoniae*	50	-	-	-	
*P. aeruginosa*	50	75	-	75	

- not determined.
